# Relationship between Bladder Cancer, Nutritional Supply, and Treatment Strategies: A Comprehensive Review

**DOI:** 10.3390/nu15173812

**Published:** 2023-08-31

**Authors:** Fan Yang, Guanmo Liu, Jiaxin Wei, Yucheng Dong, Xuebin Zhang, Yongchang Zheng

**Affiliations:** 1 Department of Urology, Peking Union Medical College Hospital, Chinese Academy of Medical Sciences and Peking Union Medical College, Beijing 100730, China; pumc_yangfan@student.pumc.edu.cn; 2 Department of Liver Surgery, Peking Union Medical College Hospital, Chinese Academy of Medical Sciences and Peking Union Medical College, Beijing 100730, China; 3 Department of Breast Surgery, Peking Union Medical College Hospital, Chinese Academy of Medical Sciences and Peking Union Medical College, Beijing 100730, China; jaisy_princeton@sina.com; 4 Department of Emergency Department, Peking Union Medical College Hospital, Chinese Academy of Medical Sciences and Peking Union Medical College, Beijing 100730, China; weijiaxin729@163.com; 5 Tsinghua Health Science Center, Chinese Academy of Medical Sciences and Peking Union Medical College, Beijing 100730, China; dongyc17@gmail.com

**Keywords:** bladder cancer, nutrient, prevention, promote recovery, therapy

## Abstract

Bladder cancer (BC) is the predominant neoplasm affecting the urinary system and ranks among the most widespread malignancies globally. The causes of bladder cancer include genetic factors; age; sex; and lifestyle factors, such as imbalanced nutrition, obesity, and metabolic disorders. The lack of proper nutrient intake leads to the development of bladder cancer because insufficient nutrients are consumed to prevent this disease. The purpose of this review was to analyze the nutrients closely linked to the onset and advancement of bladder cancer and to explore the relationship between dietary nutrients and bladder cancer. Particular emphasis was placed on nutrients that are frequently ingested in daily life, including sugar, fat, protein, and others. The focus of this research was to analyze how nutritional intake before and after surgery affects the recovery process of patients who have been diagnosed with bladder cancer. This article seeks to increase awareness among both society and the medical community about the significance of implementing appropriate dietary nutrition to reduce the chances of developing bladder cancer, enhance perioperative care for patients with bladder cancer, and aid in their recuperation.

## 1. Introduction

Urothelial carcinoma (UC) encompasses growth in the renal pelvis, ureter, bladder, and proximal urethra, with approximately 90% of cases originating from the urothelium of the bladder [1]. BC ranks as the 14th leading cause of cancer-related mortality globally [2]. Its cause is complex, involving a combination of genetic susceptibility and various lifestyle, environmental, and occupational elements, all of which may play a role in its development. Continuous progress in understanding the molecular basis of bladder cancer and its potential impact on diagnosis and treatment ensures the ongoing importance and vitality of this topic. On the other hand, the nutrients obtained through dietary intake and medication in individuals with bladder cancer are also highly significant [3].

In recent years, there has been considerable academic interest in studying the correlation between BC and comprehensive dietary patterns. A strong association between certain eating habits and bladder cancer has been observed by scientists, underscoring the significance of effectively addressing nutrition in all aspects of preventing, diagnosing, and treating bladder cancer [4,5].

The treatment recommendations of the American Society of Clinical Oncology (ASCO) Annual Meeting for muscle-invasive bladder cancer (MIBC) include neoadjuvant chemotherapy followed by radical cystectomy (RC) [6]. A significant number of MIBC patients experience a decline in nutritional status after neoadjuvant chemotherapy, which is further exacerbated by the catabolic effects of RC [7]. Bladder cancer patients may experience adverse outcomes due to malnutrition, such as sarcopenia, sarcopenic obesity, and frailty.

Advancing age is the primary risk factor for bladder cancer, with the average age of diagnosis in the United States being 73 years [8]. Individuals aged 75 and above constitute a substantial proportion of new bladder cancer cases, yet they have limited treatment options compared to younger patients. Although there are life-extending measures such as surgery and chemotherapy, numerous elderly individuals are forced to discontinue their treatment because of insufficient nourishment. Consequently, it is imperative to recognize the critical role of nutritional supplementation for older patients afflicted with bladder cancer.

According to previous epidemiological research, a reduced risk of bladder cancer has been linked to an increased intake of liquids, fruits, vegetables, yogurt, whole grains, and dietary fiber [9,10,11]. For example, apples, pomegranates, citrus fruits, cranberries, and cactus pears have the highest concentrations of anticancer components, such as phenols, flavonoids, ellagitannins, tannins, and proanthocyanidin. BC may be prevented by a sufficient supply of these natural compounds. On the other hand, consuming more meat, such as processed meat and red meat, can potentially heighten the likelihood of developing bladder cancer. However, despite these valuable findings, the specific nutrients that contribute to bladder cancer remain inadequately elucidated. This review aimed to explore the relationship between the development of bladder cancer and nutrients and the auxiliary role of nutrients in the treatment of bladder cancer and summarize the current research progress.

## 2. The Relationship between the Development of Bladder Cancer and Nutrients

**Figure 1:**
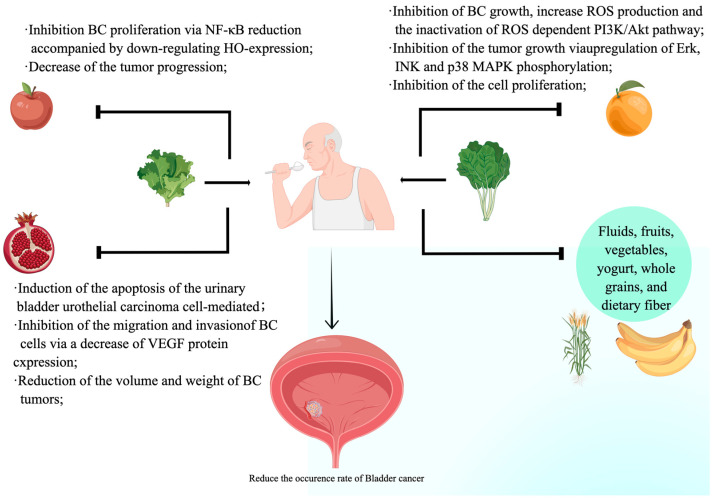
Foods rich in nutrients, including fruits and vegetables, have the effect of preventing bladder cancer: (1) numerous signaling pathways may be modulated by apples, including apoptosis, proliferation, cell growth, and mitotic catastrophe; (2) numerous signaling pathways are modulated by pomegranate, including angiogenesis, immune response, cell proliferation, glycolysis, and cell cycle, as well as apoptosis; (3) several signaling pathways may be modulated by citrus fruit, including apoptosis ROS production, growth inhibition, and cell cycle inhibition; (4) a higher intake of fluids, fruits, vegetables, yogurt, whole grains, and dietary fiber has been linked to a reduced risk of BC.

Biological plausibility supports the potential influence of dietary factors on the likelihood of bladder cancer development, as both beneficial and harmful elements of a diet are excreted through the urinary tract and directly interact with the bladder’s epithelium [12]. Several epidemiologic studies have demonstrated the importance of environmental components affecting the occurrence of this type of cancer [13], although the best-known risk factors for lower urinary tract cancer include cigarette smoke and occupational exposure to aromatic amines [14]. A higher intake of fluids, fruits, vegetables, yogurt, whole grains, and dietary fiber has been linked to a reduced risk of BC [9,10] (Figure 1).

While these findings provide valuable dietary recommendations for individuals, the nutrients or bioactive compounds responsible for the observed effects on BC risk remain to be further studied [15]. Meat intake has been suspected of being a risk factor for bladder cancer, but the results of the studies conducted thus far are contradictory [16]. Elevated rates of bladder cancer were found among subjects who had the most frequent intake of meat compared to those with the least daily intake. Po-Huang et al. studied the relationship between bladder cancer and diet. The study encompassed a cohort of 7995 Japanese-American males born between 1900 and 1919. Over a period of 22 years, 96 new cases of bladder cancer were identified. The scientists concluded that regularly eating fried vegetables, pickles, or coffee increased the chances of developing bladder cancer. However, there was no significant link found between consuming these foods and the risk of illness [17]. On the other hand, different epidemiological studies have found an inverse correlation between meat consumption and the likelihood of developing bladder cancer [18]. Increased consumption of red and processed meat was found to be a notable contributing factor for BC, resulting in a corresponding increase in the risk by 17% and 10%, respectively [19]. The increased susceptibility is probably due to the presence of N-nitroso substances, which have been proposed as potential causes of bladder cancer and are frequently present in red and processed meats [20]. The widespread exposure to environmental carcinogens from consuming processed meat and red meat should be regarded as a risk factor for bladder cancer. Future studies may potentially enable the identification of specific kinds of meat or meat-based products that offer advantageous effects.

The abundance of various antioxidants in vegetables and fruits indicates their potential as agents against cancer, thereby suggesting that consuming these food sources may effectively hinder the advancement of multiple cancer types [21]. This is primarily because a majority of these compounds and their byproducts are eliminated through urine excretion. Therefore, they interact directly with urothelial cells. Moreover, urine contains a greater amount of biologically active substances in comparison to other bodily fluids and tissues, including cancer-preventing components that may have advantageous anticancer effects [22]. According to the most recent report by the World Cancer Research Fund/American Institute for Cancer Research (WCRF/AICR), there is insufficient evidence to fully support the idea that a significant intake of fruits and vegetables can reduce the risk of bladder cancer [23]. Earlier research, which included two meta-analyses of observational studies, has indicated a link between consuming fruits and vegetables and a decreased likelihood of developing bladder cancer [24,25]. However, previous studies analyzing patient cohorts have not found any correlation between the intake of fruits and vegetables and the likelihood of developing bladder cancer [26,27]. Therefore, evidence is needed to determine whether the intake of fruits and vegetables and relative nutrients affect the advancement of BC.

Studies conducted in the past have consistently shown that a higher intake of vegetables is linked to a reduced likelihood of bladder cancer development. These investigations have focused on the influence of specific food items and food categories on the risk of bladder cancer [25,26,27]. These studies propose that this preventive effect may be attributed to the antioxidant properties of vegetables [28,29], with each additional serving of vegetables potentially resulting in a 10% reduction in bladder cancer risk. Park et al. [10] found an inverse association between the consumption of total vegetables and yellow–orange vegetables (highest vs. lowest quartile) and the risk of invasive bladder cancer among women participating in the Multiethnic Cohort Study. It is important to note that women had a slightly higher risk of developing invasive bladder cancer when comparing the second quartile to the lowest quartile of the consumption of total vegetables, light green vegetables, dark green vegetables, and cruciferous vegetables. However, the results of the present study suggest that the food group ‘vegetables and vegetable products’ could potentially be linked to the risk of BC. The responsible subgroup is still uncertain.

A comprehensive meta-analysis encompassing cohort and case-control studies observed a discernible inverse correlation between fruit consumption and susceptibility to bladder cancer. However, this association did not attain statistical significance when the meta-analysis was confined solely to cohort studies [16]. Certain fruits possess unique properties that may aid in the management and prevention of bladder cancer. Multiple preliminary studies indicate that the phytochemicals found in avocados may possess potential benefits for the prevention of cancer. For instance, investigations have demonstrated that phytochemicals and extracts derived from avocados exhibit anticarcinogenic properties, such as apoptosis induction, cell cycle arrest, antioxidant activity, and inhibition of cell proliferation, in various cancer cell lines [30,31]. Extensive documentation reports on the suppressive properties of pomegranate (*Punica granatum* L.) fruit on various forms of cancer. The outer layer (peel) of the pomegranate contains a high concentration of phenolics, flavonoids, ellagitannins (mainly punicalagin), and proanthocyanin compounds. Chang et al. conducted a study and found that an ethanol extract from the peel of pomegranates exhibited stronger inhibitory effects on the growth of T24 and J82 cells, which are human urinary bladder urothelial carcinoma cells, when compared to the extract from the pulp [32]. Furthermore, the ethyl acetate component of the ethanol extract from the peel demonstrated the highest level of inhibitory activity against urinary bladder tumors. The results of their research, which focused on bladder tumors in nude mice induced by xenografts, showed that administering the ethyl acetate layer orally at different doses (2, 5, 10, and 100 mg/kg) led to a decrease in the size and weight of T24 tumors. Additionally, it caused apoptosis in xenografted tumors and urothelial carcinoma cells. Furthermore, there are alternative food options that contain compounds that hinder the development of bladder cancer and offer guidance on diminishing the occurrence of bladder cancer. The primary components of cranberry are water, organic acids (including salicylate), fructose, vitamin C, flavonoids, anthocyanidins, catechins, and triterpenoids. An animal study involving Fischer-344 female rats demonstrated the confirmed effectiveness of cranberry juice concentrate in preventing urinary bladder cancers induced by N-butyl-*N*-(4-hydroxybutyl)-nitrosamine [33]. The anticancer properties of cranberries may involve impaired angiogenesis through the inhibition of VEGF-associated blood vessel formation, thus preventing tumor growth [34]. Avocados have a significant number of carotenoids, and studies have shown that having a higher level of carotenoids in the blood is associated with a reduced risk of bladder cancer [35]. The high level of monounsaturated fats in avocados is inversely related to the risk of bladder cancer [36]. However, Ericsson et al. found avocado consumption to be associated with a decreased risk of total cancer among men but not among women [37]. Further prospective studies should be conducted to explore this relationship. Other foods, such as citrus [27], cactus pear [34], apple [38], and others, have been suggested to prevent bladder cancer. These food studies indicate that bladder cancer can be prevented by taking in the nutrients in our food.

Evidence is scarce regarding the impact of grains and grain products on BC risk. However, Chatenoud et al. and Yu et al. both suggested that substantial consumption of whole grains might mitigate the risk of BC [39,40]. On the other hand, a recent study found that consuming a large amount of processed carbohydrate foods had a negative impact on the risk of developing BC. Therefore, it may be advantageous to conduct thorough examinations in the future, specifically focusing on whole grains and processed grain items [41].

Flavonoids, which are secondary metabolites, constitute a subgroup of polyphenols. They are widely acknowledged as the predominant polyphenols found in various sources such as fruits, chocolate, flowers, vegetables, and tea. The pharmacological implications of flavonoids have garnered significant attention due to their diverse range of effects, encompassing antioxidant, antibacterial, anti-inflammatory, cardioprotective, hepatoprotective, and anticancer properties [42,43]. In addition, they have been documented to prevent breast, colorectal, thyroid, prostate, lung, and ovarian cancers [43]. The impact of flavonoids on breast cancer has been investigated both in vivo and in vitro; however, a comprehensive analysis of these effects has not yet been conducted. Various categories of flavonoids have been found to disrupt breast cancer progression through biological pathways involving reactive oxygen species (ROS), apoptosis, ferroptosis, cancer stem cells (CSCs), epithelial–mesenchymal transition (EMT), and cell cycle arrest [44].

Several flavonoids have been shown to inhibit the proliferation and migration of bladder cancer cells, including curcumin, which has been found to induce apoptosis and repress bladder tumor growth in vitro and in vivo [45], and myricetin, which has been shown to arrest T24 bladder cancer cells at G2/M through the downregulation of cyclin B1 and the cyclin-dependent kinase cell division control protein 2 homolog [46]. Additionally, Myricetin exhibits inhibitory effects on the migration of bladder cancer cells through the downregulation of matrix metalloproteinase (MMP)-9 expression [46]. Levels of MMPs, a family of zinc-dependent ECM endopeptidases, have been shown to be significantly elevated in metastatic human cancers [47]. Moreover, Naringenin, a significant phytochemical, is classified within the flavanone category of polyphenols. It is predominantly present in citrus fruits, such as grapefruits, as well as in other sources like tomatoes, cherries, and food derived from medicinal plants [48]. Liao et al. discovered that naringenin was a novel MMP-2 inhibitor that inhibited bladder cancer cell migration and, thus, might have the potential to suppress bladder cancer metastasis [49]. Genistein, an isoflavonoid predominantly present in soybean products, was initially recognized as a suppressor of tyrosine protein kinases [50]. Although a number of beneficial actions of genistein are known, studies on the anti-cancer activity have been most extensively carried out [51,52]. Furthermore, the possibility of genistein on growth inhibitory activity in bladder cancer cells has been proposed [53,54]. Park et al. demonstrated that genistein has anti-cancer effects via the mediation of apoptotic cell death associated with G2/M arrest of the cell cycle in human urinary bladder carcinoma T24 cells [55].

Moreover, cranberry is known to possess various flavonoids, with quercetin 3-O-galactoside being the predominant compound in fruit powder [33]. Quercetin has been extensively studied for its potential anti-cancer effects [56,57]. Although quercetin 3-O-glucoside has demonstrated strong anti-proliferative activity in several bladder cancer cell lines, quercetin 3-O-galactoside did not exhibit significant inhibitory effects on cell growth [58]. 

## 3. Effects of Nutrients on Bladder Cancer Progression

**Figure 2:**
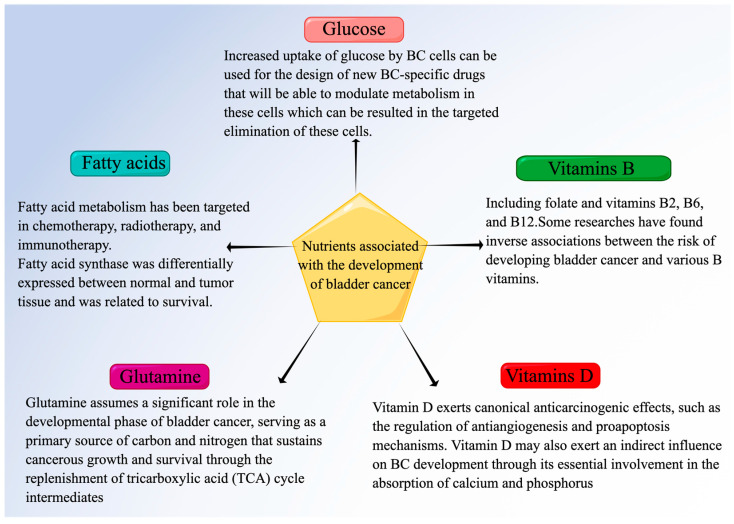
The preventive and therapeutic effects of some important nutrients on bladder cancer. (1) BC cells’ increased glucose uptake can be used for the development of new BC-specific drugs that will modulate metabolism in these cells and lead to their targeted elimination. (2) Chemotherapy, radiotherapy, and immunotherapy target fatty acid metabolism. (3) Glutamine plays a crucial role during the developmental stage of bladder cancer by acting as a principal provider of carbon and nitrogen, which supports the growth and survival of cancer cells through the replenishment of tricarboxylic acid (TCA) cycle intermediates. (4) Intake of vitamin B is associated with a reduced risk of bladder cancer.

Tumor cell biological processes are directly influenced by the energy-providing role of glucose and the molecular activities of proteins. Glucose and amino acid metabolism have attracted much attention. The tricarboxylic acid cycle facilitates the interaction between glucose, proteins, and fatty acids, resulting in a tightly integrated metabolism in tumors (Figure 2).

When the patient’s UC tumor samples were assessed, glucose levels were markedly lower in comparison to the normal urothelium [59]. Compared to untransformed urothelial cells, human UC cell lines exhibit higher glucose absorption and generate more pyruvate and lactate [60]. The tumor microenvironment (TME) plays a crucial role in shaping tumor characteristics and influencing how tumors respond to treatment. The UC experiences an increase in glucose consumption and lactate production due to a hypoxic TME, which relies on oxygen for this up-regulation. UC patients’ glucose absorption is identified through positron emission tomography/computed tomography (PET/CT), a technique that employs radioactively labeled fluorodeoxyglucose (18F-FDG), an analogous form of glucose, to visualize primary tumors and metastases. This enables the clinical use of 18F-fluorodeoxyglucose PET (18F-FDG-PET) as a precise and reliable diagnostic and prognostic tool [61]. The utilization of glucose by BC cells can be utilized for the advancement of NMR-based imaging techniques specific to BC. Furthermore, these changes in metabolism observed in BC cells can be utilized to develop novel drugs specifically targeting BC cells, ultimately leading to their targeted eradication.

Fatty acids, which are the primary byproducts of lipid metabolism, play a role in the onset and progression of metabolic disorders and cancer [62]. Chemotherapy, radiotherapy, and immunotherapy have all focused on targeting the metabolism of fatty acids (FAM). Only a few studies have examined the associations between fat, oil, and their products and BC risk and were summarized in a systematic review. This review demonstrated a positive correlation between total fat intake and breast cancer risk, based on the amalgamation of findings from three case-control studies. Conversely, no such correlation was detected in cohort studies [63]. Furthermore, specific discoveries have revealed the notable participation of Clusterin-controlled lipid synthesis in the maintenance of bladder cancer development and survival [64]. Clusterin is a target of the antidiabetic drug metformin. Metformin specifically focuses on Clusterin and successfully hinders the function of SREBP-1c, consequently suppressing the subsequent objective fatty acid synthase (FASN). By hindering the de novo production of fatty acids, this mechanism ultimately obstructs the progression of bladder cancer [64]. Lipids are crucial in signal transduction and cellular membrane synthesis. Qiao Xiong et al. constructed a prognostic model and identified key FAM genes in BC [65]. Signal transduction and cellular membrane synthesis heavily rely on lipids. They developed a predictive model and discovered important FAM genes in breast cancer (BC50). The researchers discovered that there was a differential expression of FASN between normal and tumor tissue, which was associated with survival outcomes. Over the past few years, mounting proof has emphasized its crucial significance in numerous types of cancer. The primary role of this process is to combine seven malonyl-CoA molecules and one acetyl-CoA sequentially, resulting in the formation of the initial output of fatty acid synthesis, specifically palmitate [66]. By describing the potential molecular mechanism whereby FASN functions, they provided support for further interventions using this target gene.

Cancer cells rely on glutamine, an essential nutrient, for their survival and growth. The dependence of cancerous cells on glutamine metabolism offers a chance to potentially exploit it as a target for anticancer treatment. Moreover, glutamine plays a crucial part in the progression stage of bladder cancer by acting as a main supplier of carbon and nitrogen, which supports the growth and survival of cancer cells by replenishing tricarboxylic acid (TCA) cycle intermediates [67,68]. Because of insufficient blood supply to the tumor sites and the high absorption by tumor cells, glutamine often becomes deficient in the tumor microenvironment [69]. Hence, cancer cells frequently employ tactics to endure when faced with a lack of glutamine. These cancer cells demonstrate a remarkable instance of their exceptional metabolic adaptability. In the absence of glucose, RT4 cells, which are part of urothelial bladder cancer (UBC) lines, demonstrate regular proliferation. Nonetheless, in the absence of glutamine, their growth is completely stunted, even though glucose makes up 68% of their usual dietary intake, whereas glutamine only represents 13% [70]. Wang et al. conducted a study that revealed that glutamine deprivation led to an upregulation of PD-L1 expression in bladder cancer cells, thereby facilitating the survival of cancer cells in unfavorable conditions. Nevertheless, given the importance of glutamine in the production of amino acids, the upregulation of PD-L1 is diminished in cases of prolonged glutamine deprivation. T cell production of IFN-γ may be reduced due to the increase in PD-L1 expression caused by glutamine deprivation. As a result, they suggest that the increase in PD-L1 expression in bladder cancer cells when deprived of glutamine acts to hinder the function of T cells and avoid immune detection in the face of significant nutritional limitations [71]. Furthermore, Greta et al. also used RT4 cells belonging to UBC lines, and they found the activated OxPhos metabolism determines a higher consumption of glutamine, branched-chain amino acids (BCAAs), and serine. However, when the activity of PDH and the expression of SCO2 decreased, the lower activity of OxPhos affected the serine’s catabolism, explaining the lower excretion of formate and glycine in RT cells [72].

Various food sources, such as cereals, meat, fruits, and vegetables, contain a rich abundance of B-group vitamins, including folate, as well as vitamins B2, B6, and B12. Folate, also known as vitamin B9, is a type of water-soluble vitamin that is eliminated from the body through the urinary system. These vitamins play crucial roles in essential cellular functions, particularly in the metabolism of macronutrients that provide energy [73,74]. Moreover, they play a crucial role in the one-carbon metabolic pathway, making a significant contribution to the synthesis, repair, and methylation of DNA, potentially influencing the development of carcinogenesis [75]. Only a limited number of studies have methodically evaluated the connections between B-group vitamins and the risk of UC. A recent investigation revealed clear connections between global DNA methylation and the risk of different subtypes of UC [76]. A study carried out in Spain involved 912 individuals who had been diagnosed with bladder cancer and were paired with controls from hospitals. The research results also showed that there were opposite connections between the likelihood of developing bladder cancer and different B vitamins, such as vitamin B12, vitamin B6, and vitamin B2, and a potentially important connection with folate [77]. In a meta-analysis conducted in 2014, it was observed that there may be a negative correlation between folate consumption and the risk of bladder cancer. However, this correlation was only observed in retrospective studies and not in prospective studies [78]. A prominent case-control study carried out in the United States, included in the previously mentioned meta-analysis, yielded no substantial correlation between folate consumption and the likelihood of developing bladder cancer. Nevertheless, a potential negative correlation was detected in relation to the consumption of vitamin B12 [79]. A study has found that the intake of milk, which is rich in vitamin B2 and B12, is associated with a reduced risk of bladder cancer [80]. However, Brinkman et al. and Dugué et al. found no correlation between vitamin B6 and B12 and bladder cancer [78,81]. 

According to the research findings of Mondul et al., the immune system’s overall effectiveness is significantly improved by vitamin D [82]. Furthermore, scientific evidence supports the idea that vitamin D has established anticancer properties, including the regulation of antiangiogenesis and proapoptosis mechanisms [82,83]. In relation to BC, laboratory experiments have shown that the consumption of vitamin D plays a crucial part in preserving the integrity of epithelial cells, indicating its vital contribution to the progression of BC [84]. Furthermore, it has been noted that the dynamic form of vitamin D demonstrates inhibitory impacts on migration and infiltration in human BC cell lines [85]. Nevertheless, there is a limited and inconclusive body of evidence derived from observational research concerning the influence of vitamin D on the likelihood of BC. While most studies did not discover a link between vitamin D and the risk of BC, meta-analyses have indicated a detrimental connection between the intake of vitamin D and the risk of BC, showing a consistent pattern of response to dosage [86]. Furthermore, apart from its direct effect on BC, vitamin D might also have an indirect impact on BC growth by playing a crucial role in the absorption of calcium and phosphorus [87]. Inadequate amounts of vitamin D cause a decrease in the absorption of these minerals in the intestines, leading to hypocalcemia. Considering the proven ability of calcium to protect against the formation of cancer, the occurrence of hypocalcemia might play a role in the initiation of BC. Nevertheless, despite the presumed safeguarding benefits of vitamin D, the investigation carried out by Boot et al. No significant inverse correlation was found between the intake of vitamin D through diet and the risk of BC [88]. The hypothesized protective effect of vitamin D could not be confirmed in a meta-analysis conducted by Chen et al. as well, which specifically examined the role of vitamin D intake from both diet and supplements [86]. 

## 4. Perioperative Nutrition for Bladder Cancer Patients

The majority of individuals diagnosed with bladder cancer are typically older, physically weakened, vulnerable, and experience various additional health conditions [89]. As a result of these risk factors and the complexity of the required surgery, radical cystectomy (RC), carries a significant burden [90]. Furthermore, patients frequently undergo various treatments, such as extensive surgical procedures, that additionally worsen their physical condition. Insufficient wound healing and an increased occurrence of postoperative infections have been associated with malnutrition, a notable risk factor before surgery. The significant outcomes of undernourishment are remarkable; nevertheless, they can be efficiently alleviated through dietary therapy before and after surgery. Consequently, nutritional supplementation has emerged as a fundamental component in the care of surgical patients suffering from malnutrition [91,92]. People who have been diagnosed with bladder cancer may face significant difficulties in terms of their nutrition. Moreover, older adults exhibit greater susceptibility to malnutrition in comparison to their younger counterparts. The difference can be explained by the presence of other health conditions, restricted availability of nutritious food, and reduced desire for and intake of food. In the context of cancer, people might undergo malnourishment and a decrease in body weight caused by different elements, including inadequate food intake caused by tumor-induced loss of appetite, the catabolic impact of tumors, reduced eating due to the negative effects of radiotherapy or chemotherapy, and diminished intake due to pain, anxiety, or depression. Malnourished patients undergoing cystectomy have been shown to experience a 22% increase in complication rates, with up to 15% of them developing infections due to the presence of nutritional deficiency [93,94]. These findings align with previous studies on radical cystectomy that have observed increased morbidity and mortality rates among malnourished individuals [95,96]. Moreover, individuals who were previously in good nutritional condition may be susceptible to malnutrition as a result of the substantial physiological reaction to the stress induced by surgery [97]. Consequently, implementing a peri-operative strategy for nutritional support appears to be a desirable course of action.

In the present context, nutrition support refers to the supply of nutrients to people other than the standard provision of nourishment, to enhance or sustain nutrient consumption. Nutrition support can be delivered through supplementary food or beverages, fortified food, oral nutrition supplements, enteral feeds (formulations administered via a tube into the gastrointestinal tract), or parenteral nutrition (PN) (feeds infused directly into a vein) [98]. In early studies, the utilization of PN following surgery demonstrated a notable decrease in the duration of hospitalization when compared to individuals solely administered 5% dextrose. This outcome could potentially be attributed to a diminished rate of postoperative physical activity recovery in those receiving dextrose [99]. Subsequently, a meta-analysis conducted in 2001 examined 27 randomized controlled trials (RCTs) and determined that PN administration may lead to a decrease in complication rates, albeit not mortality rates [100]. 

**Figure 3:**
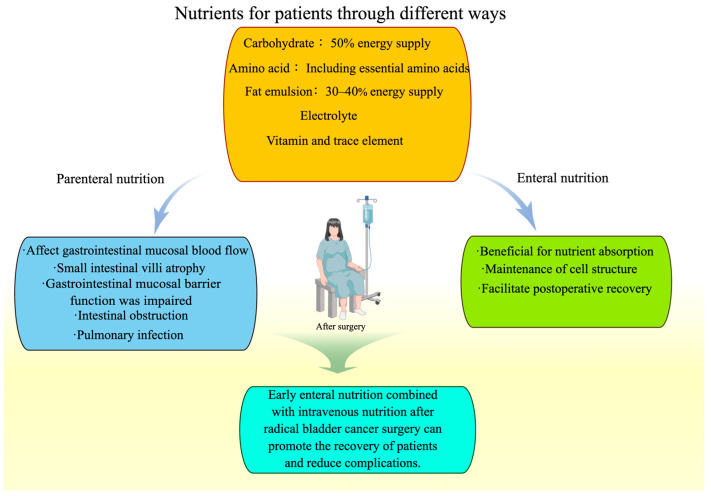
Nutrients can be delivered to patients post-bladder cancer surgery through enteral nutrition and parenteral nutrition. Parenteral nutrition is associated with numerous complications that hinder patient recovery. By complementing enteral nutrition, patient recovery can be facilitated and nutrient absorption can be enhanced.

In recent years, a range of nutritional approaches has been implemented in multimodal interventions for individuals undergoing RC [101,102]. The incorporation of early oral nutrition in multimodal interventions for RC patients is linked with decreased postoperative pain, enhanced mobilization, and a shorter duration until defecation in comparison to PN [103] (Figure 3). Nevertheless, adherence to all aspects of multimodal programs has been questioned [104]. There is also an interest in oncology surgery for prehabilitation, wherein nutrition plays a significant role as a component of multimodal preoperative treatments [105]. Multimodal prehabilitation is a comprehensive preoperative conditioning program that encompasses exercise training, nutritional therapy, and psychological interventions [106]. An increasing body of empirical research substantiates the efficacy of prehabilitation as a viable approach to mitigate perioperative functional decline [107,108,109]. Minnella et al. conducted a randomized controlled trial to assess the feasibility and efficacy of a multimodal prehabilitation program, encompassing nutritional care, exercise, and relaxation techniques, in enhancing functional capacity pre- and post-radical cystectomy. The trial involved a comprehensive dietary intervention, adhering to the validated international guideline, to achieve macronutrient balance and a daily protein intake of 1.5 g/kg. Consequently, this study offers initial evidence regarding the effectiveness of prehabilitation in reducing postoperative functional decline among patients undergoing radical cystectomy [110].

Enhanced recovery after surgery (ERAS), also known as multi-disciplinary fast-track surgery, has been a significant contributor to perioperative recovery management since the 1990s [111]. ERAS encompasses contemporary anesthesia, minimally invasive techniques, optimal analgesia, and proactive postoperative rehabilitation involving early ambulation mobilization and oral nutrition. Integration of these strategies effectively mitigates the stress response and organ dysfunction, resulting in a substantial reduction in the duration of recovery [112]. The ERAS protocol has been specifically developed to enhance postoperative recovery by promoting accelerated peristalsis, early resumption of oral intake and ambulation, and potentially reducing the length of hospital stay. ERAS pathways have been introduced recently in urology [113]. Pooled data analysis revealed that ERAS resulted in more rapid restoration of bowel function and a reduced length of hospital stay (LOS) compared to conventional recovery after surgery (CRAS) in patients after cystectomy [114]. In the study conducted by Lin et al., it was observed that the median time for bowel movement in the ERAS group was 12 h shorter than that in the CRAS group among patients undergoing RC. Additionally, the ERAS group exhibited expedited recovery in terms of fluid and regular diet tolerance, as well as ambulation [115]. Maffezzini et al. also reported that bladder cancer patients who underwent a multi-modal perioperative plan had a significantly shorter median time to recover from a regular diet (4 days) compared to those in the conventional group without a multi-modal plan (7 days) [116]. 

Immunonutrition (IM), also known as immune-boosting or immune-altering treatment, is another name for immune-enhancing or immune-modulating therapy. We discovered that, although IM is well-established in gastrointestinal surgery, there is a lack of current research on the utilization of IM in bladder tumors. Providing nutritional assistance is considered essential for patients suffering from malnutrition, and the use of IM can offer additional immune enhancement to their therapy. Boosting the immune system with a specialized intramuscular drink may provide a potentially powerful and safe method to protect against postoperative infections after an RC [117]. The present data strongly emphasize the importance of directing nutritional interventions starting from one week prior to the surgical procedure until the reestablishment of a regular diet post-surgery, to achieve optimal nutritional and immunological outcomes [118]. IM involves the incorporation of a higher quantity of nutrients that have demonstrated the ability to regulate immunological pathways [119]. These nutrients encompass glutamine, arginine, diverse amino acids, omega-3 polyunsaturated long-chain fatty acids (n-3 PUFAs), nucleotides, and antioxidants. To investigate the impact of fish oils and the amino acid arginine on peri-operative outcomes, a comprehensive review of the literature was carried out [120]. The results showed that the addition of arginine significantly decreased the occurrence of infections, complications related to wounds, and the duration of hospitalization. Following surgical trauma, the levels of arginine, which acts as a crucial precursor for nitric oxide, a potent regulator of cardiovascular stability, tend to decline. It is believed that the addition of arginine can boost the immune response at the cellular level and aid in the healing of wounds [92,121]. Furthermore, in instances involving total TPN, the administration of glutamine-TPN blends has been observed [92,119]. Glutamine, which is typically present in ample quantities within and surrounding cells, plays a vital role in sustaining the cellular milieu and facilitating swift cell division through energy provision [121]. It serves as the primary energy source for enterocytes and enhances the response of activated leucocytes [121,122]. It is recognized that following surgical stress, the body’s requirement for glutamine may surpass its inherent cellular synthesis. Consequently, there has been a recent surge in recognition regarding the potential of glutamine therapies in safeguarding gut mucosa [121]. Alivizatos et al. conducted a study involving 29 patients who underwent major abdominal surgery to compare the effects of glutamine-TPN and enteral IM on postoperative infectious outcomes [123]. The results indicated that glutamine-TPN was not inferior to enteral IM in this regard. Glutamine-TPN is particularly useful in early postoperative IM regimes when patients are unable to tolerate enteral feeding. Roth et al. found that TPN IM (Nutriflex) resulted in significantly higher protein levels (prealbumin and total protein) 7 days after surgery compared to a control group receiving Ringer’s lactate [94]. Nevertheless, this inconsistency was resolved by the twelfth day after the surgery. Nevertheless, the efficacy of glutamine-TPN after surgery still requires validation through extensive, meticulously planned RCTs that compare glutamine with both standard IM and standard TPN.

## 5. Conclusions

A substantial body of literature suggests that increased consumption of red meat, processed meat, roast meat, pork, and whole fats may be linked to an elevated risk of BC. Conversely, the consumption of fruits, vegetables, and whole grains is associated with a reduced risk of BC. Furthermore, numerous observational studies have demonstrated the potential preventive effects of vitamin B and vitamin D in BC, with particular emphasis on vitamin D’s role in inhibiting tumor angiogenesis and regulating the tumor microenvironment. However, it is imperative to validate the results of numerous observational studies through extensive randomized clinical trials to definitively address the matter and establish comprehensive guidelines and recommendations. The potential association among nutrient intake, metabolic abnormalities, and heightened susceptibility to bladder cancer, as well as their impact on the progression and management of the disease, underscores the utmost significance of prudent nutrient intake and effective management in the prevention, treatment, and rehabilitation of bladder cancer. However, present research on the correlation between bladder cancer and nutrients is limited, necessitating further investigation to comprehensively examine this domain.

## Data Availability

Not applicable due to data privacy.

## Abbreviations

American Society of Clinical Oncology, ASCO; Bladder cancer, BC; Conventional recovery after surgery, CRAS; Cancer stem cells, CSCs; Enhanced recovery after surgery, ERAS; Epithelial–mesenchymal transition, EMT; Fatty acid metabolism, FAM; Fatty acid synthase, FASN; Immunonutrition, IM; Length of hospital stay, LOS. Muscle-invasive bladder cancer, MIBC; Parenteral nutrition, PN; Positron emission tomography/computed tomography, PET/CT; Radical cystectomy, RC; Randomized controlled trials, RCTs; Reactive oxygen species; ROS; Tricarboxylic acid, TCA; The tumor microenvironment, TME; The World Cancer Research Fund/American Institute for Cancer Research, WCRF/AICR; Urothelial bladder cancer, UBC; Urothelial carcinoma, UC.
